# Stability of Signaling Pathways during Aging—A Boolean Network Approach

**DOI:** 10.3390/biology6040046

**Published:** 2017-12-18

**Authors:** Julian Daniel Schwab, Lea Siegle, Silke Daniela Kühlwein, Michael Kühl, Hans Armin Kestler

**Affiliations:** 1 Institute of Medical Systems Biology, Ulm University, 89069 Ulm, Germany; julian.schwab@uni-ulm.de (J.D.S.); lea.siegle@uni-ulm.de (L.S.); silke.kuehlwein@uni-ulm.de (S.D.K.); 2 International Graduate School of Molecular Medicine, Ulm University, 89069 Ulm, Germany; 3 Institute of Biochemistry and Molecular Biology, Ulm University, 89069 Ulm, Germany; michael.kuehl@uni-ulm.de

**Keywords:** Boolean networks, aging, reconstruction, stability

## Abstract

Biological pathways are thought to be robust against a variety of internal and external perturbations. Fail-safe mechanisms allow for compensation of perturbations to maintain the characteristic function of a pathway. Pathways can undergo changes during aging, which may lead to changes in their stability. Less stable or less robust pathways may be consequential to or increase the susceptibility of the development of diseases. Among others, NF-κB signaling is a crucial pathway in the process of aging. The NF-κB system is involved in the immune response and dealing with various internal and external stresses. Boolean networks as models of biological pathways allow for simulation of signaling behavior. They can help to identify which proposed mechanisms are biologically representative and which ones function but do not mirror physical processes—for instance, changes of signaling pathways during the aging process. Boolean networks can be inferred from time-series of gene expression data. This allows us to get insights into the changes of behavior of pathways such as NF-κB signaling in aged organisms in comparison to young ones.

## 1. Introduction

Systems Biology, the study of complex biological systems, is an emerging field in science. Many different disciplines like biology, chemistry, physics and computer sciences among others are employed to analyze biological systems. Traditional life science follows a reductionist approach. This approach has successfully identified many components and their interactions [1]. However, it does not describe system properties emerging from the interactions of these components. In contrast, Systems Biology focuses on the integration of whole biological systems. In Systems Biology, dynamic models allow for simulation of the behavior of such systems. The simulation gives valuable insights into the behavior of complex systems and hypotheses about the system can be extracted [2]. Boolean networks are one kind of dynamic models that can be used to simulate, for instance, signaling pathways [3].

Aging is a highly complex biological process which impacts health-related quality of life and life expectancy. It is characterized as the inability of tissues to maintain homeostasis [4]. Several theories have been postulated concerning the cause of aging. On a cellular level aging is, for instance, provoked by DNA damage, protein aggregation or cellular differentiation [5,6,7,8,9]. As a consequence, aging is commonly accompanied by a plethora of aging-related diseases such as cancer, neurodegeneration, diabetes, osteoporosis and cardiovascular diseases [10]. Thus, a better understanding of the underlying pathways regulating life span serves as a basis for establishing therapy concepts for age-related diseases.

In this manuscript, Boolean network reconstruction and the resulting Boolean networks are used to get insights into the changes a pathway undergoes during the process of aging. The NF-κB signaling network is analyzed with respect to changes in its susceptibility to perturbations during aging using Boolean networks inferred from gene expression data.

The transcription factor NF-κB and its intracellular signaling pathway are critical factors in muscle homeostasis [11]. Several homeostatic responses such as autophagy, apoptosis and tissue atrophy are regulated by the NF-κB signaling pathway [12]. NF-κB modulation is considered to be a factor that influences aging [13,14].

In this manuscript a time-series gene expression dataset of healthy male human muscle samples [15] was used for inferring Boolean networks. The samples in the dataset can be divided into two different stages of aging: one group of samples between 21 and 27 years and the other group between 67 and 75 years of age. The real-valued expression data is first binarized and then used to infer Boolean networks. The Boolean networks of the young and the aged phenotype are analyzed to determine their ability to maintain their function under perturbed conditions. The ability to mount an effective response to environmental and cellular stressors may play an important role in determining the onset and progression of late-life disease and aging [16].

## 2. Results

**Figure 1:**
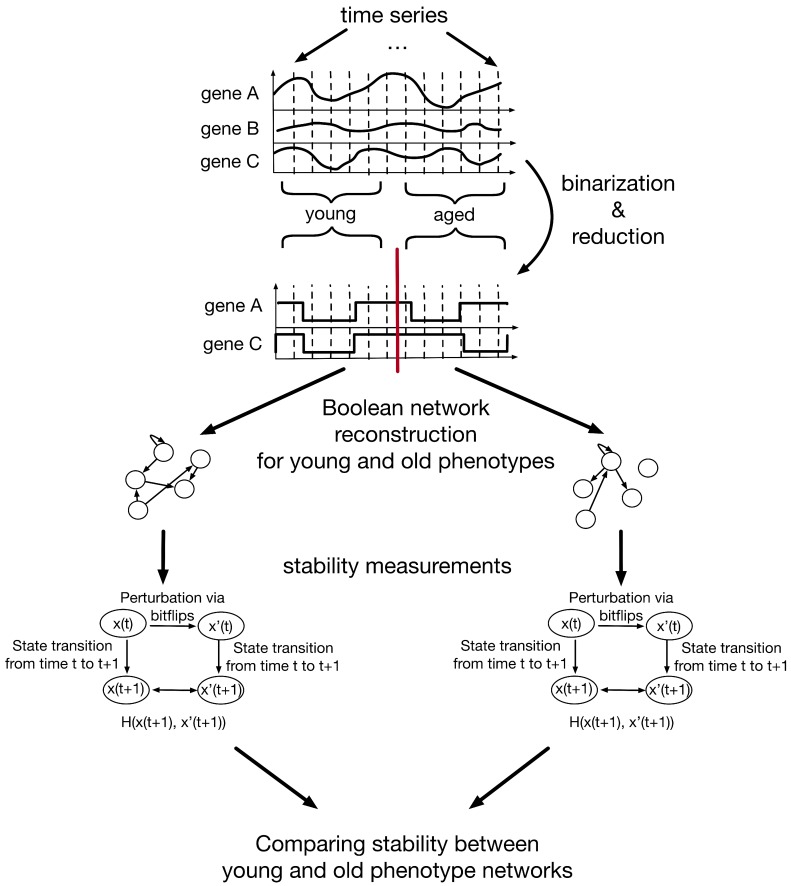
Schematic representation of a Boolean network approach to investigate stability changes in aging signaling networks. First, the time-series data is binarized and reduced using the BASC A algorithm of the R-package BiTrinA [20]. The resulting time-series data is split into two age groups (young (*n* = 7) and aged (*n* = 8)) and used to infer Boolean networks using the R-package BoolNet [21]. In the next step, the stability of the resulting Boolean networks is investigated by perturbation experiments. The best-fit algorithm can return a number of different Boolean functions for each gene in the network. From these possible functions 1000 synchronous Boolean networks are created for each age group by randomly drawing one of the inferred Boolean functions for each gene. Next, randomly generated states (x(t)) are perturbed using bitflips ($x^{\prime }\left(t\right)$). The normalized Hamming distance ($H(x,x^{\prime })$) of the successor states x(t+1) and $x^{\prime }\left(t+1\right)$ and x(t+5) and $x^{\prime }\left(t+5\right)$ of x(t) and $x^{\prime }\left(t\right)$ is computed. This is repeated for 1000 random states, the successor states of 1000 random states and random attractor state following 1000 random states with random bitflips. Finally, the mean normalized Hamming distance of these 3000 tests for each of the 1000 networks of each phenotype is compared.

The method to investigate the changing stability during aging via Boolean networks can be separated into several steps (Figure 1). In the following, these steps and their results are explained in detail.

### 2.1. Experimental Settings

#### 2.1.1. Data Processing

A publically available dataset (NCBI GEO ID GSE362) of human expression data containing samples (muscle tissue) of 15 healthy human males between 21 and 75 years of age was used. The samples were divided into two groups: the young (21 to 27 year-olds) and the aged (67 to 75 year-olds) group.

To preprocess the dataset the Affymetrix probes were mapped to Entrez IDs using the R-package biomaRt [17]. Multiple Affymetrix samples which matched to the same Entrez ID were averaged using geometric mean. Relevant genes of NF-κB signaling were selected and extracted according to the NF-κB signaling pathway in the KEGG database (95 genes; [18]). 86 of these 95 genes could be found in the aforementioned human gene expression dataset. The gene expression values of these 86 genes were selected and binarized using the BASC A algorithm [19,20]. Binarization was done using the same threshold for all samples. To reduce the size of the Boolean networks all genes that were significantly binarizable (*p* < 0.05) according to BASC’s significance test were used for network reconstruction. This resulted in a set of 22 genes: TAB1 (Entrez ID 10454), CHUK (1147), CSNK2A2 (1459), ERC1 (23085), CARD10 (29775), IRAK1 (3654), LBP (3929), LTA (4049), LTBR (4055), PLCG2 (5336), PRKCB (5579), PRKCQ (5588), PTGS2 (5743), BTK (695), TNF (7124), TRAF1 (7185), TRAF3 (7187), TRAF5 (7188), TNFSF11 (8600), BCL10 (8915), CD14 (929), CD40 (958). After binarization, the data was divided into the two different groups and the Boolean networks for NF-κB signaling of each age group was inferred using the best-fit algorithm.

#### 2.1.2. Inferring Boolean Networks from Binarized Time-Series Data

**Figure 2:**
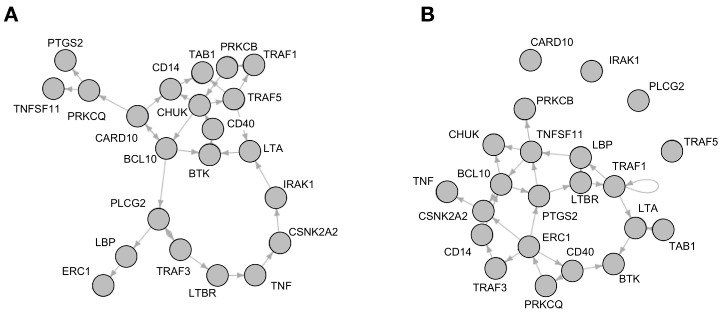
Network wiring of reconstructed Boolean networks, showing one of the possible combinations of the reconstructed functions which were drawn. (**A**) shows a network representing the young phenotype and (**B**) the aged phenotype.

The best-fit algorithm [22] as implemented in the R-Package BoolNet [21] returned a number of Boolean functions which explain the time-series data for each gene in the network—similar to a probabilistic Boolean network with equal probability for each function of one gene. For both the young and the old phenotype, each gene was reconstructed with a number of possible function variations. The functions could be reconstructed without any errors. An adjacency matrix displaying all dependencies for each component can be found in the supplement (Table S1). The network of the young phenotype shows a total number of 158 different dependencies, the network of the aged phenotype 125. These dependencies were validated by comparison to the interaction database STRING DB (www.string-db.org; [23]). In this database direct or indirect connections for all reconstructed dependencies could be found (Table S2). In the STRING DB query only experimentally verified interactions or interactions from curated databases were taken into account. In order to get synchronous Boolean networks with one function per gene, 1000 networks with randomly sampled functions were created for each of the age group networks. Figure 2 shows one example of such a synchronous Boolean network for each of the two groups.

### 2.2. Stability Measure of Boolean Networks

Biological pathways need to be fail-safe and robust against internal and external perturbations [24] in order to maintain their characteristic function. In this manuscript a number of different measurements were performed to investigate how the stability of a pathway changes during the process of aging.

The best-fit algorithm as implemented in the BoolNet package returns a number of possible Boolean functions for each gene of the pathway. All these functions have equal probability to represent the dependencies in the pathway. The number of constant genes was measured as well. A gene that is constantly set to ON/OFF is over/below the binarization threshold. If a gene is only ON/OFF in one of the age groups it can be concluded that it is differentially expressed in the different age groups.

The stability of a Boolean network can be measured by perturbing the network and then comparing the simulation results of the perturbed network with those of the original network. A network that is stable against perturbations is expected to have more similar simulation results compared to the original network than a less stable network.

Perturbation can be simulated using a random, temporary bitflip in the current state x(t) of a network. This bitflip corresponds to a temporary, punctual node shift in the network. Next, a state transition is applied to the original state x(t) and the perturbed state $x^{\prime }\left(t\right)$. The successor state $x^{\prime }\left(t+1\right)$ of $x^{\prime }\left(t\right)$ can be compared to the successor state of the original state x(t+1). The number of differing bits in $x^{\prime }\left(t+1\right)$ in comparison to x(t+1) can be measured using the normalized Hamming distance $H_{n}$. $H_{n}$ between two Boolean vectors of size *n* is here defined as:

(1) $$H_{n}\left(x,x^{\prime }\right):=\frac{1}{n}\sum_{i=1}^{n}\left|x_{i}-x_{i}^{\prime }\right|$$

A smaller, normalized Hamming distance can be linked to a more stable network. In chaotic networks a perturbation spreads exponentially throughout the network whereas in close-to-chaotic networks the perturbation spreads algebraically [25]. The more noise resistant a network is the less a perturbation is able to spread [25]. Therefore, we evaluated the Boolean networks by measuring the mean normalized Hamming distance of 1000 randomly drawn states after perturbation of one and five state transitions.

#### Analysis of Reconstructed Boolean Networks

**Figure 3:**
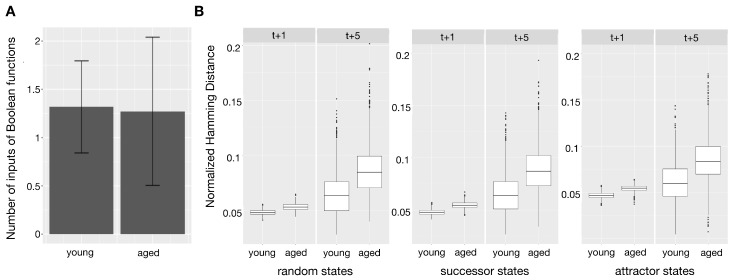
(**A**) shows the mean of the number of inputs of all Boolean functions of the young and aged phenotype Boolean networks as a bar plot. The standard deviations are included as error bars. (**B**) The boxplot shows the average, normalized Hamming distance between the successor states t+1 and t+5 of 1000 random states, the successor states of 1000 random states, attractor states following on 1000 random states and their perturbed versions for 1000 random combinations of inferred Boolean functions of the young and aged phenotype (Wilcoxon rank sum test $p<2.2\times 10^{-16}$ for all robustness comparisons).

The resulting Boolean networks for the young and the aged phenotypes were analyzed according to their robustness against perturbations. These perturbations are bitflips of randomly drawn states of the network. For this analysis, 1000 random combinations of Boolean functions which the best-fit algorithm returned for both the young and the aged network were drawn - one function for each gene. For the resulting Boolean networks, one and five state transitions based on the synchronous update scheme were applied on the initially drawn state and the perturbed state. The difference in the resulting states was then measured using the normalized Hamming distance. This was performed for each network using 1000 random initial states, a set of successor states of another 1000 randomly drawn states and a third set of random attractor states of the attractors resulting from 1000 randomly drawn initial states. These three different sets of states were analyzed as not every state in the Boolean network is equally plausible in the biological context. Results of these different kinds of perturbed states are shown in Figure 3. The number of perturbed bits was set to one. For each group of start states (random, successor, attractor) the average resulting Hamming distance for each of the 1000 networks was returned.

### 2.3. Boolean Functions

As mentioned above, the Boolean functions of each gene were derived from time-series in a data-driven way. Each of the genes in the young phenotype was reconstructed with one to up to 59 possible functions (17 possible functions per gene in the mean; data not shown). In case of four genes (TNF receptor associated factor 5 (TRAF5), Phospholipase gamma 2 (PLCγ2), Interleukin-1 receptor-associated kinase 1 (IRAK1), and Caspase recruitment domain-containing protein 10 (CARD10)) the reconstruction algorithm revealed a Boolean function which was constantly set to zero in the aged phenotype. These genes are all involved in the activation or upregulation of NF-κB [26,27,28,29]. The other 18 genes were reconstructed with one to up to 39 possible functions (seven in mean). However, the mean number of inputs per function for the reconstructed young networks (1.32) and old networks (1.27) was nearly equal (Figure 3A).

### 2.4. Network Stability

As can be seen in Figure 3B the variation in the Hamming distances between the perturbed and the original states increases with each transition in both age groups. However, in the aged group the increase in distance is more distinct. The mean Hamming distance of 0.048 (random states, rnd), 0.048 (successor states, succ) and 0.047 (attractor states, attr) in the young phenotype compared to 0.053 (rnd), 0.055 (succ) and 0.054 (attr) in the aged phenotype is an increase of roughly 15 percent for each of the settings after one state transition. The mean Hamming distance after five state transitions is increased in both age groups. However, the difference in the mean robustness of the different age groups is even higher. The mean Hamming distance in the young phenotype networks is 0.063 (rnd), 0.064 (succ) and 0.060 (attr) after five state transitions. In the aged phenotype the mean Hamming distance after five transitions is 0.085 (rnd), 0.087 (succ) and 0.083 (attr). The increase of the mean Hamming distance from the young to the old phenotype grows from about 15% after one state transition to about 35% after five state transitions (see Table 1).

## 3. Discussion

In this manuscript we analyzed binarized time-series data from high-throughput experiments using Boolean networks. Both the network reconstruction and the analysis of the resulting Boolean networks indicate differences between the two age groups. The results show that both the network representing the young phenotype and the one representing the aged phenotype were reconstructed with about the same mean input for each gene (Figure 3A). However, the interconnections between different genes vary. The aged phenotype shows some constant genes (always OFF) in contrast to the young phenotype. As these genes are not connected to other nodes by regulatory interactions they cannot be influenced by the network itself. These unregulated, constant genes are: TRAF5, PLCγ2, IRAK1 and CARD10.

NF-κB is involved in immune response. Both Tumor necrosis factor alpha (TNFα) and Interleukin 1 (IL-1) are known inducers of fever and inflammation in immune responses. TRAF5 [26] as well as PLCγ2 [27] are downstream targets of TNF receptor 1, whereas IRAK1 is a transcription factor which upregulates NF-κB in response to cellular stimulation with IL-1 [28]. Antigen contact with immunogenic substances such as lysophosphatidic acid (LPA) leads to activation of NF-κB through PLCγ2 and CARD10 [29]. According to the binarization results these factors are constantly expressed below threshold in the aged phenotype. This correlates to Welle et al. [15]: here, the authors stated a lower activity of a variety of genes in the aged group of the same dataset.

Besides the interactions, the number of different dependencies which were found by the reconstruction algorithm (158 in young phenotype, 125 in aged phenotype networks) also is decreased in the aged phenotype. The stability measurements show differing results for the networks of the two age groups. Using bitflips to perturb the network behavior is supposed to simulate internal stresses such as genotoxic stress. After perturbation, the Hamming distance was measured after one (t+1) and five (t+5) state transitions. Based on these two measurements it can be analyzed wether the perturbation spreads or the network goes back to normal after one or more state transitions. Not all states in a Boolean network might be of the same biological relevance. Thus, the perturbation experiments were examined starting from three different types of states. In addition to randomly drawn states, successors of randomly drawn states and attractor states following randomly drawn states were used in the analysis. The results of these different setups barely differ as can be seen in Table 1. On average, perturbations in the aged networks have a stronger effect on network behavior compared to the young networks: while the mean Hamming distance of both groups is increased after five state transitions (from about 15 to about 30%), the difference in the mean robustness is even higher in the young group compared to the aged group.

The number of inputs influencing each Boolean function is higher in the young phenotype, but only by a small margin. This means that the nodes in the aged phenotype are as well connected as in the young phenotype. Taken together, we can conclude that the decline in stability of the aged phenotype represented by the normalized Hamming distance is not due to a reduction in the number of input nodes. Thus, one conclusion could be that the aging NF-κB signaling pathway is less robust against internal stresses in the aged group due to a lack of redundancy in the Boolean functions.

Robustness of biological systems, for instance in the context of aging, is an emerging research topic [30,31,32]. Even though these results were based on a relatively small time-series, they show that Boolean networks can be helpful tools that allow for analysis of robustness of signaling pathways against various stresses.

For future work we plan to collect larger datasets with more time steps to increase the network reconstruction quality and to further investigate the robustness of biological pathways using Boolean networks.

## 4. Conclusions

Aging has been analyzed with Boolean networks, usually by recreating biological networks from literature and then evaluating the network patterns: e.g., Albert and Othmer [33] studied *Drosophila* embryonic development in a single network, and Herrmann et al. [34] designed a network which recreated mouse heart development. In this paper we introduce—to the best of our knowledge—a new combination of methods to create and analyze Boolean network models in the context of aging. Network construction of our models is data-driven instead of literature-based to ensure a bias-free and balanced Boolean function generation. Multiple models are constructed and then stability and expression of the models are compared. It is known that the systemic regulation of signaling pathways changes with aging. This can be seen in our data: not only is the model created based on the expression profile of the aged group less stable and robust but also some nodes of the aged model are not regulated from within the model anymore.

## 5. Materials and Methods

### 5.1. Data

**Figure 4:**
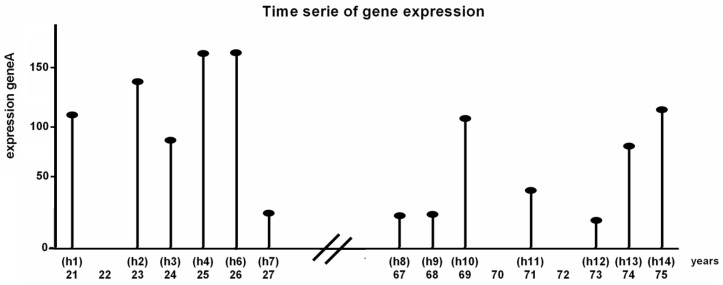
Schematic representation of one gene in the gene expression data (NCBI GEO ID GSE362). In the experiments muscle samples from 15 healthy humans of different age (21–75) were taken. The samples were arranged in ascending order by age to form a time-series. Samples of all humans between 21–27 years represent the young phenotype. The samples of all humans between 67–75 years represent the aged phenotype.

For the analysis described in this manuscript a dataset of human expression data (Affymetrix Human Genome U133A Array, 22,215 samples, obtained from NCBI GEO with ID GSE362) [15] was used. The samples were obtained from muscle cells from 15 healthy human males. These humans were divided into two groups: one group of seven humans between 21–27 years (young group) and one group of eight humans between 67 and 75 years (aged group). The samples of all 21, 23, 24, 25, 26 and 27 years old humans were used as time-series of the young group. Samples of 67, 68, 69, 71, 73, 74, 75 years old humans were used for the aged group (Figure 4). The dataset contains two samples of age 25 and two samples of age 69 which were averaged and used as one sample for each age.

### 5.2. Boolean Networks

Models are simplified representations of a real-world system aiming to mimic essential features of such a system. The models are dynamic as they describe how the systems properties change over time. There is a wide spectrum of different dynamic models. Ordinal differential equations allow for modeling the concentration rates of components like proteins [35]. Modeling differential equations for a biological system requires detailed knowledge about kinetic parameters. Often this kind of data is not available. Boolean networks are another approach to model biological processes. These models can be built when only qualitative knowledge is available. Regulatory factors of the system such as genes are represented by Boolean variables which can be either TRUE or FALSE [2,36]. The dependencies between different components of the system are described by Boolean functions. There are three major types of Boolean networks - synchronous [2,36], asynchronous [37] and probabilistic [38]. Both, synchronous and asynchronous Boolean networks are comprised of a set of Boolean variables $X=\left\{x_{1},\ldots ,x_{n}\right\},x_{i}\in B$ and the corresponding Boolean functions $F=\left\{f_{1},\ldots ,f_{n}\right\},f_{i}:B^{n}\to B$. In synchronous Boolean networks all components of the system are updated at the same time while in asynchronous Boolean networks only one component is updated at each time step. In probabilistic Boolean networks each component $x_{i}$ has a number of corresponding transition functions each of which is applied at their own probability $\left(f_{i1},p_{i1}\right),\ldots ,\left(f_{im},p_{im}\right),f_{i}:B^{n}\to B,\sum_{m}p_{im}=1$ [38]. Updates in probabilistic Boolean networks are performed synchronously after one of the possible functions for each component is selected by chance. Although asynchronous and probabilistic networks may be closer to the biological behavior both update schemes need additional assumptions in comparison to synchronous Boolean networks. Asynchronous Boolean networks have different strategies to choose which factor to update. In probabilistic Boolean networks it is necessary to determine the probability of the different transition functions [35]. This work is based on synchronous updates as they can be performed without any additional knowledge or parameters. Albeit their simplistic setup, synchronous Boolean networks proved to be valid models for various regulatory networks in different species and tissues [33,34,39,40,41,42].

As Boolean networks are dynamic models, their behavior over time is analyzed. The state of the network $x\left(t\right)=(x_{1}\left(t\right),\ldots ,x_{n}\left(t\right))$ is defined by the value of all regulatory factors $x_{i}$ at time *t*. In synchronous Boolean networks a transition to the next point in time t+1 is performed by applying all the transition functions simultaneously. This results in the successor state $x\left(t+1\right)=(f_{1}\left(x\left(t\right)\right),\ldots ,f_{n}\left(x\left(t\right)\right))$ of x(t). The dynamics of a Boolean network can be represented by a state transition graph where nodes denote states and edges the transitions from one state to another. A Boolean network with *n* regulatory factors has $2^{n}$ possible states.

Due to their finite number of states Boolean networks eventually converge on recurrent cycles of states after a number of state transitions. These recurrent states - so-called attractors - describe the long-term behavior of Boolean networks. Attractors are of special interest as they are often assumed to correspond to biological phenotypes [36,43]. All states which lead to the same attractor are associated with its so-called basin of attraction [44]. The dynamics of a Boolean network vary greatly due to the state transitions and the underlying Boolean functions. The dynamics of Boolean networks are still an active research field, e.g. temporal extensions of synchronous Boolean networks that allow to express processes on different time scales [45], model-checking-based methods for attractor identification [46] or the identification of stable states and subspaces in Boolean networks [47,48].

### 5.3. Inferring Boolean Networks

One approach to create Boolean network models is to automatically infer Boolean functions from time-series data such as gene expression data. Similar to our inference approach, a time-series of metagenomic sequencing data has been used to create a Boolean network for the gut microbiome [49] as well as measured concentration changes over time were used to construct a model of drug metabolism in leukemia [50]. Inferring Boolean networks from time-series data can be separated into two major steps: binarization and reconstruction. First the data is binarized and second the Boolean transition functions are extracted from the binarized time-series.

#### 5.3.1. Binarization of Time-Series Data

There is a variety of algorithms to binarize time-series data, for example different cluster-based approaches [51]. In this work the BASC A algorithm as proposed by Hopfensitz et al. [19] was used. The algorithm uses a series of step functions to get a robust binarization threshold. The binarization process starts by rearranging the input values in an ascending order to generate an initial step function. Next, step functions with fewer discontinuities are calculated using a dynamic programming approach. The aim is to minimize the Euclidean distance to the initial step function. Afterwards, the data was binarized by applying a threshold based on the strongest discontinuities in the step function. For a fully detailed description the reader is referred to Hopfensitz et al. [19].

#### 5.3.2. Inferring Boolean Functions

There are various types of algorithms to extract knowledge about the dependencies of the regulatory factors from time-series data. These algorithms are based on correlation [52] and Fourier transformation [53]. An algorithm to infer Boolean functions from binarized time-series data was given by Lähdismäki et al. [22]. In our work the implementation of this best-fit algorithm [22] in the R-Package BoolNet [21] was used. We briefly describe the procedure in the following. The algorithm searches for $X^{\prime }\subseteq \left\{x_{1},\ldots ,x_{n}\right\},\left|X^{\prime }\right|=k\leq n$ regulatory factors that explain $x_{i}$ with the least error and a Boolean function that is in line with the observations in the data. Finding a network which is consistent with the observations given in a time-series dataset is known as the consistency problem [54]. Solving this problem means to establish a Boolean function *f* which correctly separates true and false examples given in the data [22]. This is done by partially defined Boolean functions (pdBf). These functions denote the set of examples which are true ($T=\{X^{\prime }\left(t\right)\in B^{n}:x_{i}\left(t+1\right)=1\}$) or false $F=\{X^{\prime }\left(t\right)\in B^{n}:x_{i}\left(t+1\right)=0\}$. All pairs of $X^{\prime }\left(t\right)$ and $x_{i}\left(t+1\right)$ are extracted from the given time-series and added to *T* or *F*. Next, the error size ϵ=|F∩T| is defined by the number of inconsistencies in the pdBf. The algorithm chooses $X^{\prime }$ with the least error. To determine consistent Boolean functions truth tables are generated. Here, a Boolean function *f* is represented as a truth table indexed from 1 to $2^{n-1}$. The *i*th element of *f* is one of {1,0,*,?}, where ? means undefined and * indicates a conflict. The truth table is then filled by iterating through all examples s∈F∪T over all time steps j=1,…,m. The truth table is updated as follows :

$$f_{i}^{j}=\left\{\begin{array}{cc} 0 & \mathrm{if}s\in F\wedge f_{i}^{j-1}=?\\ 1 & \mathrm{if}s\in T\wedge f_{i}^{j-1}=?\\ {}* & \mathrm{otherwise}. \end{array}\right.$$

where *j* is the index of current time step. $f^{0}=\left(?,\ldots ,?\right)$. $i=1,\ldots ,2^{n-1}$ is the index in the truth table. The algorithm returns all Boolean functions for each component which recreate the time-series.

## Figures and Tables

**Table 1:** Overview over the measured normalized Hamming distances of the young and aged phenotypes starting from random initial states, random successor states and random attractor states compared to perturbed networks after one and after five state transitions.

	After One State Transition						After Five State Transitions					
	Young Phenotype			Aged Phenotype			Young Phenotype			Aged Phenotype		
	Min	Max	Mean	Min	Max	Mean	Min	Max	Mean	Min	Max	Mean
random initial state	0.041	0.056	0.048	0.044	0.065	0.053	0.028	0.151	0.063	0.040	0.201	0.085
random successor state	0.041	0.057	0.048	0.045	0.067	0.055	0.027	0.143	0.064	0.034	0.209	0.087
random attractor state	0.036	0.057	0.047	0.037	0.064	0.054	0.004	0.144	0.060	0.007	0.211	0.083

## Supplementary Materials

The following are available online at www.mdpi.com/2079-7737/6/4/46/s1, Table S1: Adjacency matrices of BNs, Table S2: Interactions from STRING DB.
